# Potential mechanisms of the fatigue‐reducing effect of cognitive‐behavioral therapy in cancer survivors: Three randomized controlled trials

**DOI:** 10.1002/pon.5710

**Published:** 2021-05-03

**Authors:** Fabiola Müller, Feri Wijayanto, Harriët Abrahams, Marieke Gielissen, Hetty Prinsen, Annemarie Braamse, Hanneke W.M. van Laarhoven, Perry Groot, Tom Heskes, Hans Knoop

**Affiliations:** ^1^ Department of Medical Psychology Amsterdam University Medical Centers University of Amsterdam Amsterdam Public Health Research Institute Amsterdam The Netherlands; ^2^ Department of Health Psychology University of Groningen University Medical Center Groningen Groningen The Netherlands; ^3^ Faculty of Science, School of Psychology The University of Sydney Sydney Australia; ^4^ Institute for Computing and Information Sciences Radboud University Nijmegen The Netherlands; ^5^ Department of Informatics Universitas Islam Indonesia Yogyakarta Indonesia; ^6^ Academy Het Dorp Arnhem The Netherlands; ^7^ Siza (disability service) Arnhem Arnhem The Netherlands; ^8^ Department of Medical Oncology Radboud University Nijmegen The Netherlands; ^9^ Department of Medical Oncology Cancer Center Amsterdam Amsterdam University Medical Centers University of Amsterdam Amsterdam The Netherlands; ^10^ Department of Medical Psychology Amsterdam University Medical Centers Expert Center for Chronic Fatigue Vrije Universiteit Amsterdam Amsterdam The Netherlands

**Keywords:** cancer, cancer‐related fatigue, catastrophizing, causal modeling, cognitive‐behavioral therapy, depression, mediation, oncology, psycho‐oncology, randomized controlled trial

## Abstract

**Objective:**

Fatigue is a common symptom among cancer survivors that can be successfully treated with cognitive‐behavioral therapy (CBT). Insights into the working mechanisms of CBT are currently limited. The aim of this study was to investigate whether improvements in targeted cognitive‐behavioral variables and reduced depressive symptoms mediate the fatigue‐reducing effect of CBT.

**Methods:**

We pooled data from three randomized controlled trials that tested the efficacy of CBT to reduce severe fatigue. In all three trials, fatigue severity (checklist individual strength) decreased significantly following CBT. Assessments were conducted pre‐treatment and 6 months later. Classical mediation analysis testing a pre‐specified model was conducted and its results compared to those of causal discovery, an explorative data‐driven approach testing all possible causal associations and retaining the most likely model.

**Results:**

Data from 250 cancer survivors (*n* = 129 CBT, *n* = 121 waitlist) were analyzed. Classical mediation analysis suggests that increased self‐efficacy and decreased fatigue catastrophizing, focusing on symptoms, perceived problems with activity and depressive symptoms mediate the reduction of fatigue brought by CBT. Conversely, causal discovery and post‐hoc analyses indicate that fatigue acts as mediator, not outcome, of changes in cognitions, sleep disturbance and depressive symptoms.

**Conclusions:**

Cognitions, sleep disturbance and depressive symptoms improve during CBT. When assessed pre‐ and post‐treatment, fatigue acts as a mediator, not outcome, of these improvements. It seems likely that the working mechanism of CBT is not a one‐way causal effect but a dynamic reciprocal process. Trials integrating intermittent assessments are needed to shed light on these mechanisms and inform optimization of CBT.

## 1 INTRODUCTION

Cancer‐related fatigue is a distressing symptom that persists in around 25% of cancer survivors long after completion of their cancer treatment.^1^, ^2^ Cognitive‐behavioral therapy (CBT) is an evidence‐based intervention for reducing cancer‐related fatigue.^3^ It is based on the cognitive‐behavioral model of fatigue, stating that cancer and its treatment initially *precipitate* fatigue, while cognitive‐behavioral variables *perpetuate* fatigue.^4^ CBT is a modular treatment targeting these cognitions and behaviors, specifically: dysfunctional cognitions regarding fatigue, difficulties coping with cancer and cancer treatment, high fear of cancer recurrence, a fluctuating or low activity pattern, deregulated sleep‐wake rhythm, and perceived poor social support. Changes in the targeted cognitive‐behavioral variables are assumed to explain the beneficial effect of CBT.

In line with the cognitive‐behavioral model of fatigue, recent studies among cancer patients undergoing treatment for chronic myeloid leukemia^5^ and advanced cancer^6^ suggest that increased self‐efficacy, reductions in helplessness and focusing on symptoms act as mediators of the fatigue‐reducing effect brought by CBT. The role of physical activity is less clear. Studies among cancer survivors^7^, ^8^, ^9^ and patients on active treatment^10^ found no evidence that increased physical activity, assessed *objectively*, explained the fatigue‐reducing effect brought by CBT. The study by Abrahams and colleagues,^7^ however, found that increases in patients' *self‐reported* activity had a mediating effect. Studies on the putative mediating effect of other cognitive‐behavioral variables that are thought to maintain fatigue in cancer survivors, and are targeted in CBT, are currently lacking.

Next to changes in cognitive‐behavioral variables, a reduction in depressive symptoms may also be a working mechanism of CBT. Symptoms of depression are prevalent among cancer survivors^11^ and commonly co‐occur with cancer‐related fatigue.^2^, ^12^ Notably, while not directly targeted in CBT for fatigue, depressive symptoms were reduced^13^ and found to partially mediate the treatment effect of CBT^14^ in severely fatigued patients with multiple sclerosis and diabetes type 1, respectively. Similarly, a decrease in depressive symptoms may also mediate the effect of CBT in cancer survivors.

While providing some valuable insights, analytical limitations of the above mentioned studies preclude drawing firm conclusions about the working mechanisms of CBT. These studies tested a classical mediation model in which targeted cognitive‐behavioral variables are the pre‐specified mediators and fatigue is the pre‐specified outcome. While in line with the CBT‐treatment model, these confirmatory, theory‐driven analyses might lead to false conclusions since there are many alternative causal pathways that are not tested.^15^ In contrast, the causal discovery approach allows exploration to discover the causal associations between variables without the need to pre‐specify the mediators or outcome.^16^ Instead, all possible causal associations are tested and the most likely model is retained. Causal discovery has been shown to find consistent results which are close to the true underlying mechanism of simulated datasets.^17^, ^18^

To overcome the limitations mentioned above, the current study investigated a comprehensive set of putative mediators that might explain the fatigue‐reducing effect of CBT and compares the results from the classical mediation analysis to those of causal discovery. We expected that improvements in the cognitive‐behavioral variables thought to maintain cancer‐related fatigue and which are targeted in CBT, as well as reduced depressive symptoms, mediate the reduction in fatigue severity following CBT. For the current analyses, data of the two aforementioned trials (i.e., Gielissen‐trial,^8^, ^19^ Prinsen‐trial^9^) were combined with data of a recent trial (i.e., Abrahams‐trial^20^).

## 2 METHOD

### 2.1 Study design

Data from three randomized controlled trials (RCTs), assessing the efficacy of CBT for fatigue in severely fatigued cancer survivors, were pooled and re‐analyzed. Cancer survivors were randomly assigned to either internet‐based^20^ or face‐to‐face^9^, ^19^ CBT or a waitlist control condition. Patients in both conditions were assessed at baseline and 6 months later. In all three trials, cancer survivors randomized to CBT reported significantly lower fatigue scores at follow‐up compared to survivors in the waitlist condition.

### 2.2 Participants

Patients were eligible for participation if they had completed cancer treatment with curative intent ≥3 months^20^ or ≥12 months^9^, ^19^ previously, were severely fatigued (≥35 checklist individual strength, subscale fatigue [CIS‐fatigue]) and aged ≥18 years. Patients treated for breast cancer,^20^ various tumor types,^19^ and those with a malignant, solid tumor or a non‐Hodgkin's lymphoma^9^ were included. Patients with a comorbidity that could explain their fatigue and those who underwent psychological or psychiatric treatment were excluded. Patients included in the Abrahams‐trial assessing internet‐based CBT were also required to have internet access and possess basic internet skills. A description of the study samples is provided in Table 1.

**TABLE 1:** Descriptive statistics per trial

Characteristics		Abrahams‐trial		Gielissen‐trial		Prinsen‐trial	
		CBT‐condition *n* = 63	Control‐condition *n* = 63	CBT‐condition *n* = 43	Control‐condition *n* = 44	CBT‐condition *n* = 23	Control‐condition *n* = 14
Age in years, range		52.2 (8.3), 32–72	50.6 (7.7), 31–68	44.2 (10.1), 20–61	44.8 (10.3), 21–61	48.5 (9.2), 29–64	50.7 (10.9), 29–65
Gender, female, *n* (%)		63 (100%)	63 (100%)	20 (46.5%)	22 (50.0%)	10 (43.5%)	9 (64.3%)
Cancer type, *n* (%)							
	Breast	63 (100%)	63 (100%)	14 (32.6%)	12 (27.3%)	7 (30.4%)	6 (42.9%)
	Gynecological	‐	‐	2 (4.7%)	4 (9.1%)	1 (4.3%)	‐
	Testis	‐	‐	12 (27.9%)	12 (27.3%)	3 (13.0%)	‐
	Other	‐	‐	15 (34.9%)	16 (36.4%)	12 (52.2%)	8 (57.1%)
Treatment, *n* (%)							
	Surgery only	5 (7.9%)	2 (3.2%)	6 (14.0%)	7 (15.9%)	3 (13.0%)	1 (7.1%)
	Surgery plus RT and/or CT	58 (92.1%)	61 (96.8%)	27 (62.8%)	31 (70.5%)	18 (78.3%)	8 (57.1%)
	No surgery, only RT and/or CT	‐	‐	10 (23.3%)	6 (13.6%)	1 (4.3%)	4 (28.6%)
	Other	‐	‐	‐	‐	1 (4.3%)	1 (7.1%)
Time since treatment in months, range		38.0 (31.2), 3–166	32.4 (25.7), 3–147	66.2 (52.0), 15–219	56.1 (41.7), 13–181	52.2 (63.9), 14–329	45.3 (36.8), 12–126
Fatigue							
	Pre^a^, range	45.1 (7.0), 26–56	44.7 (7.4), 24–56	47.9 (6.6), 35–56	47.5 (6.7), 35–56	44.4 (6.2), 35–54	46.1 (4.8), 38–56
	Post, range	26.7 (11.5), 8–56	38.9 (11.1), 12–56	27.3 (14.6), 8–56	41.8 (9.7), 9–56	22.5 (10.2), 8–51	38.6 (11.8), 9–49
Self‐efficacy							
	Pre	18.8 (2.8)	18.2 (3.3)	18.4 (2.8)	18.4 (2.9)	18.6 (2.1)	18.1 (2.4)
	Post	22.7 (3.5)	19.1 (3.8)	22.5 (3.9)	18.6 (3.4)	23.5 (3.7)	18.4 (2.8)
Fatigue catastrophizing							
	Pre	21.2 (5.8)	21.4 (5.9)	18.3 (10.7)^b^	14.6 (7.8)^b^	22.5 (7.6)	19.1 (6.7)
	Post	15.2 (5.0)	19.7 (5.7)	10.6 (9.0)^b^	13.7 (9.5)^b^	14.6 (4.2)	19.3 (7.1)
Focusing on symptoms							
	Pre	31.4 (8.7)	33.0 (7.9)	Not assessed	Not assessed	32.0 (8.9)	28.6 (7.1)
	Post	18.3 (8.6)	27.1 (8.5)	Not assessed	Not assessed	19.4 (7.4)	25.6 (10.6)
Fear of cancer recurrence							
	Pre	7.5 (2.4)	8.1 (2.2)	15.2 (3.3)^b^	14.0 (3.7)^b^	8.3 (1.7)	7.7 (2.5)
						14.6 (3.8)^b^	15.6 (5.6)^b^
	Post	6.3 (1.8)	7.2 (2.1)	13.8 (4.0)^b^	14.8 (3.6)^b^	6.2 (1.9)	6.2 (2.8)
						11.3 (3.6)^b^	14.8 (5.1)^b^
Problems coping with cancer							
	Pre	13.0 (14.7)	14.4 (14.5)	12.9 (12.7)	9.6 (10.1)	12.9 (15.4)	15.4 (16.5)
	Post	9.6 (10.5)	11.1 (13.9)	7.6 (10.1)	8.2 (10.2)	6.8 (11.1)	11.9 (12.3)
Physical activity							
	Pre	70.0 (16.7)	72.1 (19.5)	69.0 (22.2)	66.0 (18.3)	65.1 (15.7)	77.5 (17.7)
	Post	72.5 (15.3)	68.4 (18.7)	73.1 (21.9)	65.1 (24.5)	73.1 (18.0)	79.9 (23.6)
Perceived problems with activity							
	Pre	14.6 (4.6)	13.6 (5.0)	15.0 (5.0)^b^	14.4 (4.7)^b^	13.7 (4.2)	14.9 (3.6)
	Post	9.3 (4.3)	12.0 (5.0)	8.2 (4.4)^b^	11.3 (4.4)^b^	8.2 (4.2)	13.4 (5.2)
Sleep disturbance							
	Pre	100.4 (66.2)	94.0 (59.2)	75.3 (65.9)	60.6 (55.3)	87.2 (69.3)	78.1 (56.4)
	Post	23.9 (34.7)	67.1 (55.9)	24.6 (36.7)	55.3 (59.8)	30.5 (46.3)	59.9 (60.3)
Problems with social support							
	Pre	11.5 (3.3)	10.7 (3.0)	11.4 (3.8)	10.5 (2.6)	10.8 (2.6)	10.3 (2.9)
	Post	10.2 (3.3)	10.5 (3.2)	10.3 (3.2)	10.9 (4.0)	10.2 (2.3)	10.6 (2.8)
Depressive symptoms							
	Pre	6.5 (3.6)	6.7 (3.4)	2.4 (2.8)^b^	1.4 (1.7)^b^	6.6 (4.1)	5.4 (3.5)
	Post	3.5 (3.7)	5.7 (3.7)	1.3 (2.3)^b^	1.7 (1.9)^b^	2.9 (3.3)	5.2 (3.6)

*Note:* Unless otherwise indicated, values represent mean values and standard deviations.

Abbreviations: CT, chemotherapy; post, post‐assessment; pre, pre‐assessment; RT, radiotherapy.

^a^ For *n* = 3 CBT‐patients and *n* = 4 Control condition‐patients in the Abrahams‐trial, CIS‐fatigue has dropped under the cut‐off of 35 points between screening and baseline assessment. From the Gielissen‐ and Prinsen‐trial, CIS‐fatigue score of the screening were available only.

^b^ Indicates that a different questionnaire (‐version) has been used to assess the construct. For the data regarding Fear of cancer recurrence of the Prinsen‐trial, *n* = 16/8 (pre‐assessment) *n* = 7/5 (post‐assessment) patients for the CBT‐ and Control condition, respectively, completed another questionnaire‐version. The remaining patients completed th same questionnaire as in the other two trials.

As this study aims to identify working mechanisms of CBT, only data of patients with complete pre‐ and post‐fatigue assessment were included. Additionally, for patients randomized to the CBT condition, only data from patients who had some exposure to (i.e., at least started) CBT were included. Patients in the control condition in the Abrahams‐trial were asked to indicate whether they followed a fatigue intervention during the waitlist period. We excluded one patient who reported to have followed an evidence‐based fatigue intervention (i.e., mindfulness therapy).

### 2.3 Cognitive‐behavioral therapy

CBT was conducted according to the cognitive‐behavioral model of fatigue. Patients started with the module “Goal setting” and finished with the module “Realizing of goals”. The intermediate six modules coincide with the six fatigue‐perpetuating factors. Table S1 briefly outlines each module. CBT was provided by trained cognitive‐behavioral therapists, either entirely face‐to‐face^9^, ^19^ or by two initial face‐to‐face sessions followed by supervised online modules and a final face‐to‐face evaluation session.^20^ The intervention was targeted to the patient, that is, baseline scores on instruments assessing perpetuating factors of fatigue and information from the intake session were used to determine which modules patients were to follow.

### 2.4 Control condition

Patients randomized to the control condition were placed on a 6‐months waitlist for receiving CBT.

### 2.5 Outcome measure

In all three trials, the CIS was administered. Its 8‐item subscale CIS‐fatigue was used to assess *fatigue severity*. Items (e.g., “I feel tired”) refer to the past 2 weeks and are scored on a 7‐point Likert scale, ranging from (1) “Yes, that is true” to (7) “No, that is not true”. A higher score indicates more severe fatigue (range 8–56). A score of ≥35 indicates severe fatigue in cancer survivors.^21^

### 2.6 Putative mediators

Putative mediators were assessed with questionnaires (i.e., self‐efficacy, fatigue catastrophizing, focusing on symptoms, fear of cancer recurrence, problems coping with cancer, perceived problems with activity, sleep disturbance, problems with social support, depressive symptoms) and actigraphy (i.e., objective physical activity), see supplemental material for details. For four concepts, different questionnaire (‐versions) were administered among trials and one concept was not assessed in the Gielissen‐trial (see Table 1 and “Statistical analyses” for how these were handled).

### 2.7 Statistical analyses

We implemented two analytical approaches. We first conducted classical mediation analysis with the PROCESS macro (version 3.4) in SPSS.^22^ We tested a pre‐specified parallel multiple mediation model that estimates the direct and indirect effects of the predictor Condition (Control = 0, CBT = 1) on the outcome fatigue severity through 10 putative mediators (as assessed at post‐assessment). The estimate for each path accounts for the other mediation paths as well as for covariates (i.e., pre‐treatment values of fatigue severity and the putative mediators, sex, age, time since treatment). The analysis was conducted with 5000 bootstrap samples. 95% confidence intervals (CI) excluding 0 indicate significance.

We next applied causal discovery by employing Bayesian constraint‐based causal discovery (BCCD) in RUCausal package for R​.^23^ BCCD examines the complete model without pre‐specifying associations between variables and provides a reliability estimate, indicating the degree of confidence in the associations found. We integrated the below stated background knowledge to obtain a meaningful model:

1. Post‐treatment variables and Condition (Control vs. CBT) cannot cause pre‐treatment variables;
2. Condition cannot be caused by any other variable;
3. Covariates cannot be caused by any other variable.

To improve the stability of the results, we applied half‐sampling: we constructed 1000 datasets, each time sampling half of the data at random, and ran the BCCD algorithm on each of these datasets, yielding 1000 models. For these models, as recommended in the literature,^24^ we set the reliability threshold to 0.7. Next, we calculated the average of these 1000 models, for which we set the post‐bootstrap reliability threshold to 0.5. The strength of the direct associations between variables were computed by employing the bootstrapped‐model as a basis. Cohen's *f* ^2^ was used as an effect size index^25^ after ensuring that there is no effect flow via any confounding variables by performing covariate adjustment (back‐door adjustment),^26^ Table S2 a/b. *f* ^2^ ≥ 0.02 indicates a small and *f* ^2^ ≥ 0.15 a medium effect.

For both statistical approaches, to prevent losing data and to gain more statistical power, we computed z‐scores for variables that were assessed with a different questionnaire (‐version) across trials. Missing values without alternative were left as missing. In the classical mediation analysis, listwise deletion was applied, leaving 52% of the cases. In the causal analysis, pairwise deletion was applied, using an average of 92% of cases among variable pairs (range 52%–100%).

## 3 RESULTS

**FIGURE 1:**
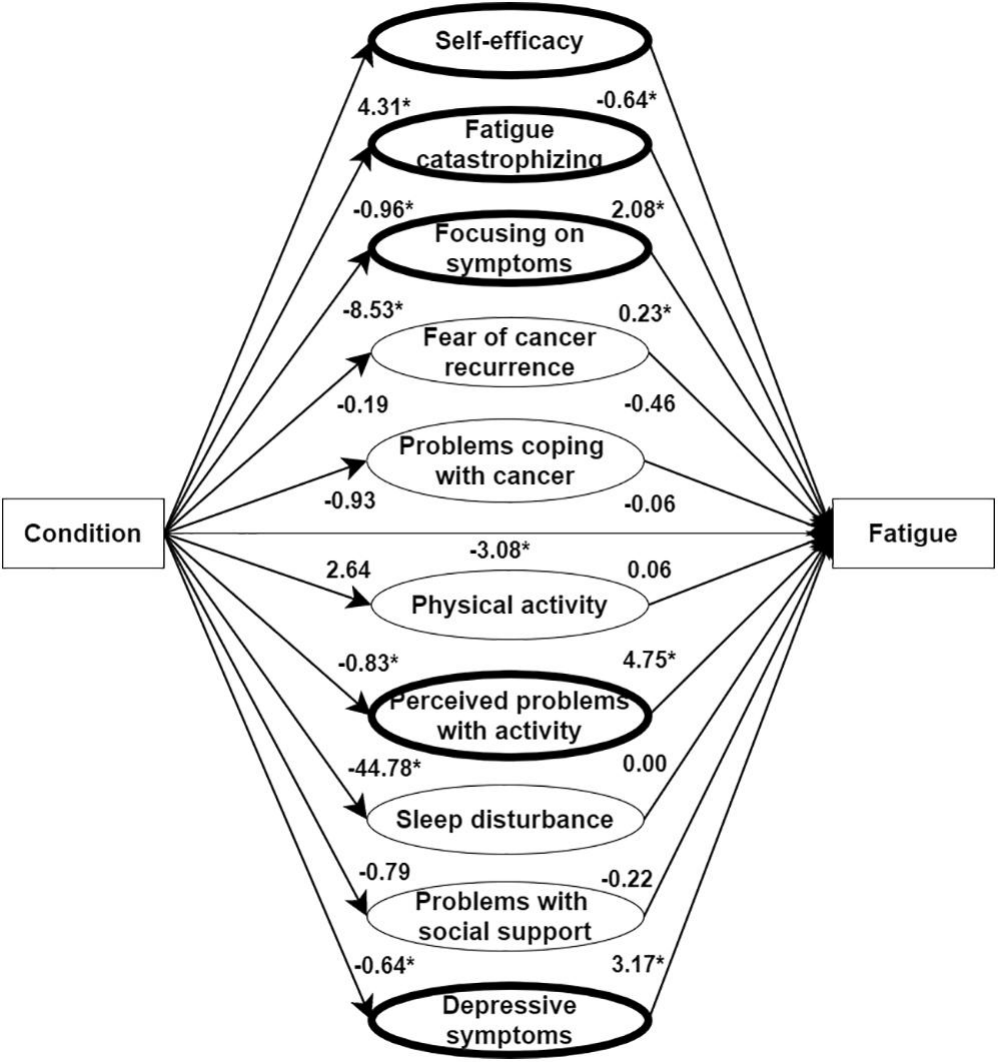
Classical mediation model (pre‐specified), *with* z‐score transformation. Values represent regression coefficients. Variables with a bold border indicate significant mediators. * indicates a significant path

Both the classical mediation analysis and the causal discovery confirmed that CBT leads to a significant reduction in fatigue. The classical mediation analysis (Figure 1) partly confirmed our expectations regarding the factors mediating the effect of CBT on fatigue: increased self‐efficacy (*ab* = −2.76, CI [−4.86, −1.01]), decreased fatigue catastrophizing (*ab* = −1.99, CI [−3.82, −0.43]), reduced focusing on symptoms (*ab* = −1.95, CI [−4.05, −0.11]), a reduction in perceived problems with activity (*ab* = −3.94, CI [−6.51, −2.09]) and lowered depressive symptoms (*ab* = −2.02, CI [−3.71, −0.46]) significantly mediated the reduction in fatigue brought by CBT. The remaining variables were not mediators of fatigue.

**FIGURE 2:**
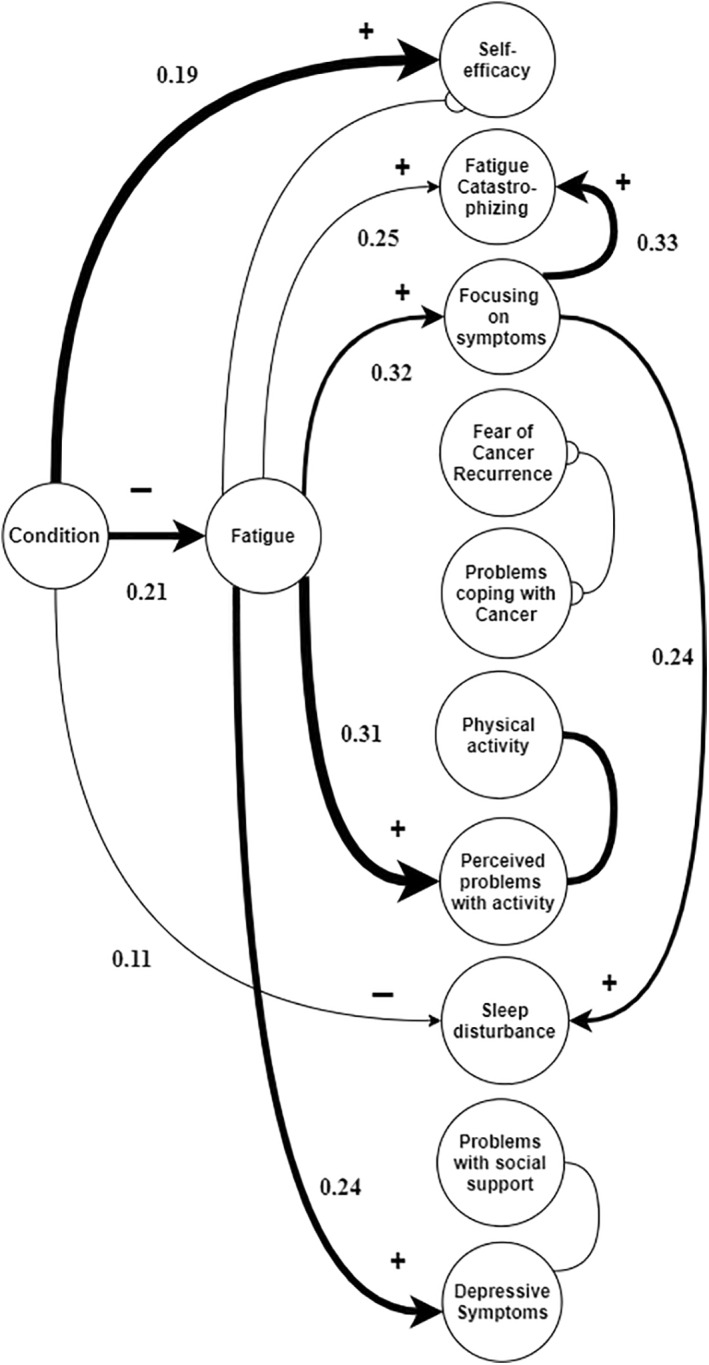
Causal discovery model (data‐driven), *with* z‐score transformation. The tail (‐) represents the origin of the causal effect and the arrowhead (➤) the direction of the causal effect. The circle (o) represents an association in which the origin and direction are unclear. The undirected lines (−) indicate the presence of selection bias (i.e., bias introduced by the sample selection). All links represent a causal association of which the edge has a post‐bootstrap reliability coefficient of ≥0.5, with a thicker line corresponding to a more likely causal association between variables. The values represent the strength of the causal effects (see also Table S2a)

In contrast, causal discovery suggested that fatigue is not an outcome but a mediator of changes in some of the putative mediators (Figure 2). CBT had a direct positive causal effect on self‐efficacy (*f* ^2^ = 0.19) and a direct negative causal effect on sleep disturbance (*f* ^2^ = 0.11) and fatigue severity (*f* ^2^ = 0.21). Fatigue severity, in turn, had a positive causal effect on fatigue catastrophizing (*f* ^2^ = 0.25), focusing on symptoms (*f* ^2^ = 0.32), perceived problems with activity (*f* ^2^ = 0.31), and depressive symptoms (*f* ^2^ = 0.24). Focusing on symptoms had a positive causal effect on fatigue catastrophizing (*f* ^2^ = 0.33) and on sleep disturbance (*f* ^2^ = 0.24). While an association between physical activity and perceived problems with activity was found, its direction could not be determined. Pre‐treatment variables were strongly associated with the corresponding post‐treatment variables. The covariates sex, age and time since treatment were unrelated to the causal process.

As the finding that fatigue might be a mediator rather than an outcome was unexpected, we conducted two post‐hoc analyses to investigate the role of fatigue in more detail. First, we performed classical mediation analyses to test our results from causal discovery. In four separate models, we pre‐specified fatigue as mediator of either changes in fatigue catastrophizing, focusing on symptoms, perceived problems with activity, and depressive symptoms. All these mediation paths were significant indicating that, as in the causal discovery analysis, fatigue may act as a mediator, not an outcome, of changes in cognitions and depressive symptoms (Figure S1). These models, with fatigue as pre‐specified mediator, were a better fit than those with fatigue as pre‐specified outcome (SEM analyses in lavaan using R, BIC as goodness‐of‐fit index^27^), Table S3. As a second post‐hoc analysis, within causal discovery, we calculated the degree of confidence for fatigue being a mediator or an outcome. This degree of confidence was defined as the minimum of the reliabilities of each of the links in a mediation path, averaged over all 1000 resampled models. Also these post‐hoc analyses favor models in which fatigue is a mediator as opposed to an outcome (Table S4).

## 4 DISCUSSION

Pooled data from three RCTs were analyzed to investigate whether changes in cognitive‐behavioral variables and in depressive symptoms explain the fatigue‐reducing effect brought by CBT in cancer survivors. The results from classical mediation analysis are partly in line with the CBT‐treatment model and our expectations. Improvements in self‐efficacy, fatigue catastrophizing, focusing on symptoms and perceived problems with activity were found to act as mediators of the reduction in fatigue. The mediating role of changes in cognitions has been demonstrated in studies among patients with various fatigue‐related conditions,^5^, ^6^, ^14^, ^28^, ^29^, ^30^ in which also confirmatory classical mediation analyses were conducted.

As a second analytical approach we applied causal discovery, which does not rely on the pre‐specification of mediational pathways. This analysis suggests that fatigue acts as a mediator, not an outcome, of changes in fatigue catastrophizing, focusing on symptoms, perceived problems with activity and, indirectly, in sleep disturbance. This finding was unexpected as it suggests a causal order opposite to that suggested in the CBT‐treatment model and the current literature. However, partly in line with the treatment model, CBT appeared to have a direct causal effect on improved self‐efficacy and reduced sleep disturbance.

Problems with social support and the two cancer‐specific variables did not mediate (classical mediation analysis) nor play any causal role (causal discovery) in the reduction of fatigue. Available data of the Abrahams‐trial show that the corresponding modules on social support (40%), fear of cancer recurrence (75%) and coping with cancer (39%) were indicated for only a subset of patients, while the other modules were indicated for all. This implies that a substantial group did not score high on these scales, which might have reduced our statistical power to find an effect.

Improvements in self‐reported problems with activity were found to be a mediator of (classical mediation analysis) or be mediated by (causal discovery) improvements in fatigue. Physical activity, objectively assessed, was neither a mediator nor mediated by fatigue. This finding was not entirely unexpected: earlier analyses with two^8^, ^9^ of the three pooled datasets and studies among other patient groups^14^, ^28^, ^31^, ^32^, ^33^ question the mediating role of *objectively* assessed physical activity while pointing towards a mediating role of patients' *perceived* activity. However, increasing physical activity has been reported by patients as one of the most helpful components of CBT for fatigue^32^ and its graded activity module has been found to lead to a greater reduction in fatigue among cancer survivors than its other modules.^7^ This suggests that actual increases in physical activity, in some way, might contribute to the fatigue‐reducing effect of CBT. It has been hypothesized that patients, at least temporarily, increase their physical activity which leads to improvements in patients' dysfunctional cognitions about their ability to become active, which in turn, explains the treatment effect.^8^, ^31^ Our data add to this evidence that points towards the important role of *perceived* activity. And while our data show that objective and perceived activity are associated, with only two assessment time points, we cannot draw conclusions about the potential role of temporarily improved *objectively* assessed activity.

Improvements in depressive symptoms were found to be a mediator of (classical mediation analysis) or be mediated by (causal discovery) improvements in fatigue. The former is in line with the aforementioned trial among patients with diabetes,^14^ in which decreased depressive symptoms, next to changes in cognitions, mediate the fatigue‐reducing effect brought by CBT. Results from causal discovery and post‐hoc analyses, again, suggest the opposite causal direction. This direction is in line with longitudinal, observational research among cancer patients showing that, over time, fatigue predicts depression, rather than depression predicting fatigue.^34^, ^35^, ^36^

While the results of causal discovery and post‐hoc analyses were unexpected, these do not necessarily invalidate the CBT‐treatment model, aimed at relieving fatigue through targeting cognitive‐behavioral variables known to perpetuate fatigue. In line with the treatment model, we found evidence for improvements in targeted cognitions and sleep disturbance throughout CBT. However, results from causal discovery and our post‐hoc analyses suggests that testing a pre‐specified model that assumes a one‐way causal effect is too simplistic to understand how CBT works. It seems more likely that the working mechanism of CBT is a dynamic reciprocal process of improvements in targeted cognitions, sleep disturbance, possibly temporarily improved objectively assessed activity, and reduced fatigue.^32^, ^37^ This reciprocal process might operate in short time‐intervals within or across days^38^ and hence might not be captured by only few widely spaced and simultaneous assessments. Improvements in depressive symptoms, which are not directly targeted in CBT and might more likely change as a result of reduced fatigue (see above), may play a different role in this process. Unraveling the possible dynamic changes induced by CBT requires study designs that integrate frequent assessments throughout treatment. Determining the appropriate frequency and spacing between assessments to capture these processes is a challenge future research should address.

### 4.1 Study limitations

Only two assessment time points were available for analyses. While causal discovery allowed us to explore the direction of the associations in a data‐driven way, we cannot unravel a possible dynamic reciprocal process. Relatedly, both analytical approaches assessed the associations in question at the group‐level. It seems likely, however, that the working mechanisms of CBT differ between individuals.^39^ Future studies integrating frequent assessments, as suggested above, can overcome both limitations. Further, our sample was predominantly female (75%) and survivors were selected based on the presence of severe fatigue. The associations found might not generalize to males or patients who are not severely fatigued. Next, while causal discovery does not rely on the pre‐specification of a mediation model, it tends to explain strong correlations through direct effects and weaker correlations through indirect effects. As CBT is designed to reduce patient fatigue, it has a strong effect on this variable. Therefore, fatigue severity and Condition were strongly correlated in our sample (Figure S2). This might explain why causal discovery suggests that fatigue severity is most likely directly, as opposed to indirectly, associated with CBT. However, both post‐hoc analyses support the notion that changes in fatigue from pre‐ to post‐treatment are more likely to act as a mediator as opposed to an outcome. Lastly, in order to retain as much data as possible, we performed z‐score transformation to four instruments. We do not expect this to have impacted our results, as our sensitivity analyses without z‐score transformation yielded comparable results to those presented here (Figure S3/S4).

### 4.2 Clinical implications

Our study does not provide the expected insights into the working mechanism of the fatigue‐reducing effect of CBT. Therefore, we cannot formulate recommendations for optimizing the treatment protocol. If future studies find evidence for the hypothesized dynamic reciprocal process that brings by the reduction in CBT, this might provide the opportunity to optimize the treatment: Close monitoring of those variables involved in the reciprocal process *throughout* CBT could help track the patient's progress and, if needed, inform how to adapt the treatment to improve its effect (e.g., intensify modules/exercises). Similarly, monitoring of those variables *after* completing CBT might help to timely signal relapse and consequently offer booster sessions.

Along fatigue, depressive symptoms and sleep disturbance form a symptom cluster that is common among cancer patients.^34^, ^40^ It is therefore encouraging that the latter two symptoms also improved after CBT, making it a candidate treatment for patients presenting with this symptom cluster.

## 5 CONCLUSIONS

In line with the CBT‐treatment model, targeted cognitions, targeted sleep disturbance and depressive symptoms improve in cancer survivors undergoing CBT for severe fatigue. Unexpectedly, fatigue acted as a mediator and not as an outcome of these improvements when assessed pre‐ and post‐treatment. These results challenge the currently predominant model relying on testing a pre‐specified one‐way causal effect to explain reduced fatigue. It is more likely that CBT unfolds its fatigue‐reducing effect through a reciprocal dynamic process between improvements in targeted cognitions, sleep disturbance, possibly temporarily improvements in objectively assessed activity, and reduced fatigue. Future trials should integrate intermittent assessments of these variables throughout the delivery of CBT to be able to disentangle its working mechanisms and derive recommendations to optimize its protocol.

## CONFLICTS OF INTEREST

The authors have no conflicts of interest to declare.

## ETHICS STATEMENT

The three RCT's were reviewed and approved by the Medical Ethical Committee of the Radboud University Medical Centre (Abrahams‐trial: 2013/167, Prinsen‐trial: 2008/200, Gielissen‐trial: CWOM 0011‐0246). The Abrahams‐trial was registered in the Dutch Trial Registry (NTR4309). The Gielissen‐trial was registered in the Dutch Trial Registry (NTR107). The Prinsen‐trial was registered at ClinicalTrials.gov (NCT01096641). All patients provided written informed consent.

## AUTHORS CONTRIBUTION

All listed authors have made a substantial contribution to the work, have been involved in the drafting and/or revising the manuscript, gave final approval for submission of this version of the manuscript and agree to be accountable for all aspects of the work.

## Supporting information

Supplementary Material

## Data Availability

The data that support the findings of this study are available from the principle investigators upon reasonable request, including a data analysis plan and research questions. Permission for data sharing will be asked from the medical ethical commission.
