# Supplementary material for: Potential mechanisms of the fatigue‐reducing effect of cognitive‐behavioral therapy in cancer survivors: Three randomized controlled trials
Source: Psychooncology. 2021 May 3;30(9):1476–84. doi: 10.1002/pon.5710 (PMC8518952; doi:10.1002/pon.5710)
Supplement: Supplementary file 1 — Supplementary Material [file PON-30-1476-s001.pdf]

Supplementary material

Description of instruments used to assesses the putative mediators

Self-efficacy was assessed with the Self-Efficacy Scale, as used in a similar study.[1] Seven items (e.g. '*I think I could positively influence my fatigue'*) assess patients' perceived control over their fatigue. Items are scored on a 4-point Likert scale ranging from (1) '*No, I am convinced that is not true'* to (4) '*Yes, I am convinced that is true*'. Higher scores indicate a higher self-efficacy regarding fatigue (range 7-28).

In the Abrahams- and Prinsen-trial, *Fatigue catastrophizing* was assessed with the Fatigue Catastrophizing Scale.[2] Ten items (e.g. '*I imagine the fatigue becoming even more intense and exhausting'*) assess patients' negative cognitions and feelings of helplessness about fatigue. Items are scored on a 5-point Likert-scale from (1) '*Never true'* to (5) '*All of the time true'* (range 10-50). In the Gielissen-trial, an instrument based on the Pain Catastrophizing Scale[3] has been administered in which 13 items are scored on a 5-point Likert-scale (range 0-52). For both instruments, higher scores indicate more catastrophizing.

In the Abrahams- and Prinsen-trial, *Focusing on fatigue* was assessed with the subscale Focusing on Symptoms of the Illness Management Questionnaire.[4] Nine items (e.g. '*I spend a lot of time thinking about my fatigue*') assess patients' focus on fatigue during the previous month. Items are scored on a 6-point Likert-scale, ranging from (1) '*Never'* to (6) '*Always'*. Higher scores indicate more focusing on fatigue (range 9–54). Focusing on fatigue was not assessed in the Gielissen-trial.

In the Abrahams-trial and, partly, the Prinsen-trial, *Fear of cancer recurrence* was assessed with three items of the Modified Cancer Acceptance Scale.[5] Items (e.g. '*I am worried about a tumor relapse*') are scored on a 4-point Likert scale ranging from (1) 'N*ot at all applicable'* to (4) '*Very applicable*' (range 3-12). In the Gielissen-trial, and partly in the Prinsen-trial, a 7-item version of the instrument has been administered (range 7-28). For both instruments, higher scores indicate higher fear of cancer recurrence.

Problems coping with cancer and cancer treatment was assessed with the Impact of Event Scale.[6,7] Fifteen items (e.g. '*I thought about it when I didn't mean to'*) assess patients' intrusive cognitions and avoidance of reminders of cancer and its treatment during the past 2 weeks. Items are scored on a 4-point scale (scoring: 0-1-3-5), ranging from (0) '*Not at all*' to (5) '*Often'*. Higher scores indicate more intrusive cognitions and more avoidance behavior (range 0-75).

Physical activity was assessed in two ways. First, in all three trials, *objective physical activity* was assessed with actigraphy. The actigraph is a motion-sensing device that gets attached to the ankle and was worn by patients for 12 consecutive days and nights at pre- and post-treatment. Twelve daily physical activity scores were calculated, expressed in the average number of accelerations per 5 minutes. An average daily level of physical activity was computed over this period with higher scores indicating more physical activity. Previous research supports the reliability and validity of the actigraph.[8] Second, in the Abrahams- and Prinsen-trial, self-reported perceived activity was assessed with the subscale CIS-activity. Three items (e.g. *'I don't do much during the day'*) assess patients' perceived problems with their daily activity over the past 2 weeks.[9] Items are scored on a 7-point Likert scale (see main

text). In the Gielissen-trial, a previous version of the CIS-activity has been administered which differs from the recent version in the wording of the three items; the scoring is identical. Higher scores indicate more *perceived problems with activity* (range 3-21).

Sleep disturbance was assessed with the subscale sleep and rest of the Sickness Impact Profile-8.[10] Seven items (e.g. '*I sleep or nap during the day*') assess patients' functional impairment in daily life regarding sleep and rest. Patients are instructed to check those items that are applicable to them on a given day and are related to their health. Scores of the checked items are weighed. Higher scores indicate more limitations in sleep and rest (range 0-499).

Problems with social support were assessed with the discrepancy subscale of the Sonderen Social Support Inventory.[11] Eight items (e.g. '*What is your opinion about the extent to which people: Stand by you*?') assess discrepancies between the amount of received and the amount of desired social support. Items are scored on a 4-point Likert scale ranging from (1) '*I miss it*' to (4) '*It happens too often*'. Higher scores indicate a higher discrepancy in support (range 8-32).

In the Abrahams- and Prinsen trial, *depressive symptoms* were assessed with the depression subscale of the Hospital Anxiety and Depression Scale.[12] Seven items (e.g. '*I feel as if I am slowed down*') are rated for the past week and are scored on a 4-point Likert scale, with differing responses per item, for example (0) '*Nearly all the time*' to (3) '*Not at all'*. In the Gielissen-trial, depressive symptoms were assessed with the Beck Depression Inventory for Primary Care,[13] a 7-item scale scored on a 4-point Likert scale. For both scales, higher scores indicate higher levels of depressive symptoms (range 0-21).

TABLE S1 Overview of the cognitive-behavioral therapy modules

Modules	Brief description of module content
**Goal setting**	• Psycho-education regarding cognitive-behavioral model of fatigue<br>• Patients set intervention goals in concrete activities that will be performed again when the patient is no longer severely fatigued
**Helpful thinking**	• Targets dysfunctional cognitions regarding fatigue<br>• Patients learn to identify unhelpful thoughts and replace them with helpful thoughts, gain more self-efficacy and to focus less on their fatigue
**Sleep-wake rhythm**	• Targets a deregulated sleep-wake rhythm<br>• Patients establish regular sleep-wake pattern and follow sleep-hygiene practices
**Social support**	• Targets low social support and negative interactions<br>• Patients learn how to communicate with significant others about their fatigue, be assertive and adapt the expectations they have from their environment
**Coping with cancer and cancer treatment**	• Targets difficulties to cope with cancer and cancer treatment<br>• Patients learn to process their cancer experience and related distress through exposure
**Fear of cancer recurrence**	• Targets high fear of cancer recurrence<br>• Patients learn to get insight into triggers of their anxiety and learn to adopt helpful cognitions through reality testing
**Graded activity**	• Targets a fluctuating or low (physical) activity pattern<br>• Patients with a fluctuating activity pattern learn to evenly distribute their activities during the day and will subsequently gradually increase their daily activity level (e.g. walking, cycling)<br>• Patients with low activity pattern immediately start with gradual increase in their daily physical activity (e.g. walking, cycling)
**Realizing goals**	• Patients make an action plan to realize their formulated interventions goals<br>• Patients learn to let go of the regular sleep-wake rhythm and even distribution of activities<br>• Patients evaluate their progress

TABLE S2a Causal discovery: Effect sizes for the model *with* z-score transformation

Causal effect	Regression equation	Coefficient	SE	f^2
**Condition to**				
Fatigue	Fatigue ~ Condition	-0.51	0.86	0.21
Self-efficacy	Self-efficacy ~ Condition	0.48	0.88	0.19
Sleep disturbance	Sleep disturbance ~ Condition	-0.35	0.93	0.11
**Fatigue to**				
Fatigue catastrophizing	Fatigue catastrophizing ~ Fatigue	0.58	0.81	0.25
Focusing on symptoms	Focusing on symptoms ~ Fatigue	0.71	0.73	0.32
Perceived problems with activity	Perceived problems with activity ~ Fatigue	0.67	0.74	0.31
Depressive symptoms	Depressive symptoms ~ Fatigue	0.55	0.83	0.24
**Focusing on symptoms to**				
Fatigue catastrophizing	Fatigue catastrophizing ~ Focusing on symptoms + Fatigue	0.45	0.72	0.33
Sleep disturbance	Sleep disturbance ~ Focusing on symptoms + Condition	0.45	0.84	0.24

The effect size Cohen's f^2 was calculated as ($\frac{R^2}{1+R^2}$). The direct effects are computed among post-treatment variables based on the causal model in Figure 2 (main text). $f^2 \geq 0.02$ indicates a small effect, $f^2 \geq 0.15$ indicates a medium effect, $f^2 \geq 0.35$ indicates a large effect.[14] SE = Standard error.

TABLE S2b Causal discovery: Effect sizes for the model *without* z-score transformation

**Causal effect**	**Regression equation**	**Coefficient**	**SE**	**f²**
**Condition to**				
Fatigue	Fatigue ~ Condition	-0.51	0.86	0.21
Self-efficacy	Self-efficacy ~ Condition	0.48	0.88	0.19
Sleep disturbance	Sleep disturbance ~ Condition	-0.35	0.94	0.11
**Fatigue to**				
Fatigue catastrophizing	Fatigue catastrophizing ~ Fatigue + Fatigue†	0.64	0.79	0.28
Focusing on symptoms	Focusing on symptoms ~ Fatigue + Fatigue†	0.72	0.73	0.32
Perceived problems with activity	Perceived problems with activity ~ Fatigue + Fatigue†	0.69	0.71	0.33
Depressive symptoms	Depressive symptoms ~ Fatigue	0.67	0.77	0.29
**Focusing on symptoms to Sleep disturbance**	Sleep disturbance ~ Focusing on symptoms + Condition	0.45	0.84	0.24
**Physical activity to Perceived problems with activity**	Perceived problems with activity ~ Physical activity	-0.50	0.87	0.17

The effect size Cohen's f^2 was calculated as ($\frac{R^2}{1+R^2}$). The direct effects are computed among post-treatment variables based on the causal model in Figure S4. $f^2 \geq 0.02$ indicates a small effect, $f^2 \geq 0.15$ indicates a medium effect, $f^2 \geq 0.35$ indicates a large effect.[14] SE = Standard error. † as assessed at the pre-treatment. The remaining variables are those assessed at post-treatment.

TABLE S3 Classical mediation post-hoc analysis: Goodness-of-fit of models with fatigue as the outcome versus fatigue as the mediator, based on models *with* z-score transformation

Putative mediator	BIC	
	**Fatigue as outcome**	**Fatigue as mediator**
**Self-efficacy**	3078	3066[†]
**Fatigue catastrophizing**	2498	2447[†]
**Focusing on symptoms**	2229	2211[†]
**Perceived problems with activity**	2546	2497[†]
**Depressive symptoms**	2531	2468[†]

BIC = Bayesian information criterion, goodness-of-fit index computed using lavaan package for R. A model with lower BIC is preferred, with a differences of > 10 (indicated by [†]) providing strong support for the model with the lower BIC value.[15]
Fatigue as an outcome refers to a mediation path of: Condition → Putative mediator → Fatigue.
Fatigue as a mediator refers to a mediation path of: Condition → Fatigue → Putative mediator.

TABLE S4 Causal discovery post-hoc analysis: The degree of confidence of mediation paths with fatigue as the outcome versus fatigue as the mediator. Based on the model *with* z-score transformation

Putative mediators	Degree of confidence	
	**Fatigue as outcome**	**Fatigue as mediator**
**Fatigue catastrophizing**	0.018	0.349
**Focusing on symptoms**	0.123	0.379
**Perceived problems with activity**	0.012	0.505
**Sleep disturbance**	0.015	0.140
**Depressive symptoms**	0.021	0.367

The values in this table provide the degree of confidence in each mediation path, defined as the reliability computed by the causal discovery algorithm BCCD (bayesian constraint-based causal discovery) for this path, averaged over 1000 runs of this algorithm on half-sampled datasets. This definition leads to conservative values which do not sum up to one (100%) since we only considered two out of many alternative mediation models.
Fatigue as an outcome refers to a mediation path of: Condition → Putative mediator → Fatigue.
Fatigue as a mediator refers to a mediation path of: Condition → Fatigue → Putative mediator.

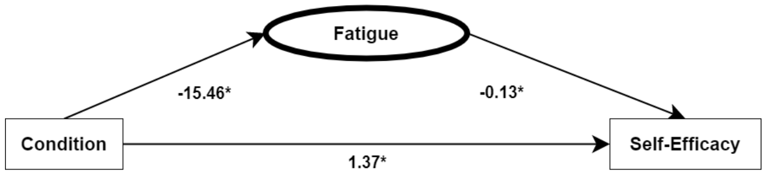

a. Classical mediation model with Fatigue as mediator and Self-efficacy as an outcome. The mediation path is significant (*ab* = 1.97, CI[0.64, 3.79]).

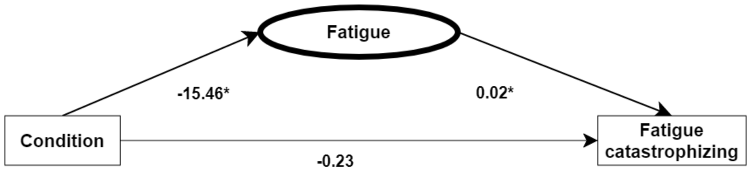

b. Classical mediation model with Fatigue as mediator and Fatigue catastrophizing as an outcome. The mediation path is significant (*ab* = -0.33, CI[-0.77, -0.06]).

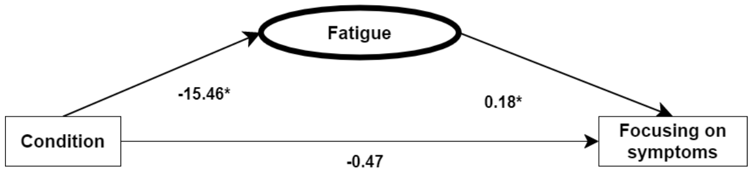

c. Classical mediation model with Fatigue as mediator and Focusing on symptoms as an outcome. The mediation path is significant (*ab* = -2.84, CI[-6.71, -0.09]).

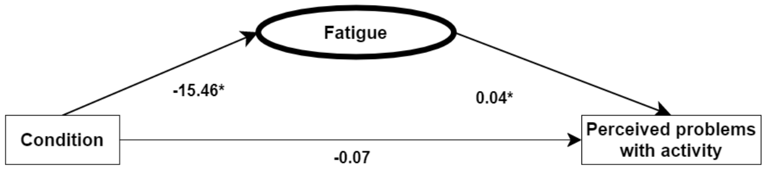

d. Classical mediation model with Fatigue as mediator and Perceived problems with activity as an outcome. The mediation path is significant (*ab* = -0.67, CI[-1.04, -0.38]).

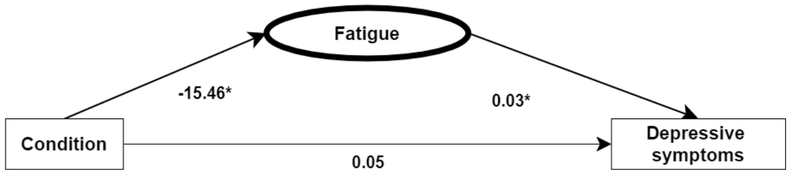

e. Classical mediation model with Fatigue as mediator and Depressive symptoms as an outcome. The mediation path is significant (*ab* = -0.42, CI[-0.81, -0.10]).

FIGURE S1a-e Classical mediation post-hoc analyses testing models with Fatigue as pre-specified mediator. Note. Along Fatigue, each model includes the other putative mediators entered as mediators. But for clarity, only Fatigue is displayed here. Models are based on scales *with* the z-score transformation. * indicates a significant path.

FIGURE S2 Correlation matrices

		M	SD	1	2	3	4	5	6	7	8	9	10	11	12	13	14	15	16	17	18	19	20	21	22	23	24	25	26
1.	**Fatigue, pre**	45.90	6.89	1.00*																									
2.	**Self-efficacy, pre**	18.44	2.89	-0.22*	1.00*																								
3.	**Fatigue Catastrophizing, pre†**	0.00	1.00	0.34*	-0.26*	1.00*																							
4.	**Focusing on Symptoms, pre**	31.85	8.32	0.30*	-0.28*	0.57*	1.00*																						
5.	**Problems Coping with Cancer, pre**	12.84	13.72	0.03	-0.09	0.39*	0.35*	1.00*																					
6.	**Physical Activity, pre**	69.59	18.76	-0.26*	0.06	-0.04	-0.12	0.07	1.00*																				
7.	**Percieved Problems with Physical Activity, pre†**	0.00	1.00	0.40*	-0.09	0.07	0.14	-0.03	-0.25*	1.00*																			
8.	**Sleep Disturbance, pre**	84.99	63.41	0.13*	-0.12	0.25*	0.17*	0.19*	-0.06	0.23*	1.00*																		
9.	**Problems with Social Support, pre**	10.98	3.14	0.03	-0.09	0.15*	0.09	0.17*	0.04	0.07	0.05	1.00*																	
10.	**Fear of Recurrence, pre†**	0.00	1.00	0.06	-0.08	0.36*	0.33*	0.58*	0.08	0.02	0.20*	0.13*	1.00*																
11.	**Depressive Symptoms, pre†**	0.00	1.00	0.21*	-0.34*	0.38*	0.34*	0.24*	-0.08	0.22*	0.20*	0.34*	0.26*	1.00*															
12.	**Condition**	0 = Control/Male		0.00	0.08	0.12	-0.05	0.01	-0.06	0.07	0.10	0.15	0.00	0.11	1.00*														
13.	**Sex**	1= CBT/Female		0.00	-0.03	0.06	0.08	0.09	-0.05	0.07	0.16	0.02	0.05	0.19*	-0.11	1.00*													
14.	**Age**	48.69	9.57	-0.11	-0.11	-0.09	-0.12	0.06	-0.12	0.05	0.11	-0.06	0.03	0.02	0.03	0.34*	1.00*												
15.	**Time since Treatment**	46.34	41.83	0.10	0.02	0.04	-0.11	-0.21*	-0.01	0.01	-0.12	0.11	-0.14*	-0.04	0.12	-0.22*	-0.06	1.00*											
16.	**Fatigue, post**	32.83	13.51	0.31*	-0.22*	0.07	0.17*	0.03	-0.09	0.06	0.16*	-0.02	0.11	0.18*	-0.59*	0.08	-0.02	0.01	1.00*										
17.	**Self-efficacy, post**	20.86	4.10	-0.20*	0.44*	-0.05	-0.15	-0.12	0.02	-0.05	-0.11	-0.10	-0.12	-0.19*	0.61*	-0.07	-0.06	0.00	-0.71*	1.00*									
18.	**Fatigue Catastrophizing, post†**	0.00	1.00	0.23*	-0.28*	0.44*	0.29*	0.20*	0.04	0.00	0.11	0.11	0.22*	0.25*	-0.39*	0.19*	0.01	0.02	0.58*	-0.53*	1.00*								
19.	**Focusing on Symptoms, post**	22.41	9.43	0.17*	-0.21*	0.30*	0.46*	0.23*	0.05	0.04	0.13	0.00	0.24*	0.22*	-0.51*	0.09	-0.13	0.01	0.69*	-0.56*	0.67*	1.00*							
20.	**Problems Coping with Cancer, post**	9.19	11.41	0.04	-0.13	0.31*	0.33*	0.62*	0.00	-0.01	0.20*	0.06	0.51*	0.19*	-0.09	0.10	0.05	-0.22*	0.22*	-0.26*	0.39*	0.38*	1.00*						
21.	**Physical Activity, post**	70.92	20.23	-0.15*	0.05	0.10	-0.04	0.11	0.56*	-0.16*	-0.04	-0.04	0.15*	-0.10	0.13	-0.06	0.05	-0.01	-0.20*	0.15*	-0.03	-0.06	-0.01	1.00*					
22.	**Percieved Problems with Physical Activity, post†**	0.00	1.00	0.31*	-0.24*	0.07	0.18*	-0.02	-0.23*	0.34*	0.10	0.00	0.04	0.25*	-0.41*	0.13	0.06	0.04	0.67*	-0.49*	0.39*	0.50*	0.10	-0.36*	1.00*				
23.	**Sleep Disturbance, post**	43.11	51.57	0.16*	-0.17*	0.10	0.15	0.07	-0.10	0.12	0.28*	0.14*	0.11	0.19*	-0.45*	0.13	-0.03	-0.03	0.49*	-0.46*	0.41*	0.53*	0.14*	-0.16*	0.38*	1.00*			
24.	**Problems with Social Support, post**	10.41	3.27	0.13*	-0.04	0.19*	0.05	0.08	-0.03	0.13	0.10	0.55*	0.07	0.24*	-0.08	0.06	-0.11	0.10	0.17*	-0.18*	0.24*	0.07	0.04	-0.02	0.21*	0.18*	1.00*		
25.	**Fear of Recurrence, post†**	0.00	1.00	0.08	-0.14*	0.34*	0.29*	0.46*	0.08	-0.08	0.10	0.06	0.66*	0.18*	-0.23*	0.10	-0.02	-0.17*	0.23*	-0.24*	0.35*	0.36*	0.53*	-0.04	0.11	0.20*	0.07	1.00*	
26.	**Depressive Symptoms, post†**	0.00	1.00	0.18*	-0.26*	0.20*	0.21*	0.19*	-0.02	0.15*	0.17*	0.26*	0.19*	0.65*	-0.27*	0.21*	0.06	0.04	0.56*	-0.50*	0.46*	0.50*	0.26*	-0.16*	0.51*	0.40*	0.37*	0.24*	1.00*

S2a Means, Standard Deviations [SDs] and correlation matrix showing the pairwise correlations between the different variables. Polychoric correlation was used to compute the correlation between the dichotomous variables Condition and Sex. Polyserial correlation was used for pairs of either Condition or Sex with other variables. Pearson correlation was used for the remaining variable pairs. † indicates variables to which *z-score* transformation has been applied, consequently their mean values = 0 and SDs = 1. * indicates a significant correlation ($p < 0.05$). pre = pre-assessment, post = post-assessment.

		M	SD	1	2	3	4	5	6	7	8	9	10	11	12	13	14	15	16	17	18	19	20	21	22	23	24	25	26
**1.**	**Fatigue, pre**	45.90	6.89	1.00*																									
**2.**	**Self-efficacy, pre**	18.44	2.89	-0.22*	1.00*																								
**3.**	**Fatigue Catastrophizing, pre**	21.28	6.22	0.33*	-0.23*	1.00*																							
**4.**	**Focusing on Symptoms, pre**	31.85	8.32	0.30*	-0.28*	0.57*	1.00*																						
**5.**	**Problems Coping with Cancer, pre**	12.84	13.72	0.03	-0.09	0.46*	0.35*	1.00*																					
**6.**	**Physical Activity, pre**	69.59	18.76	-0.26*	0.06	-0.08	-0.12	0.07	1.00*																				
**7.**	**Percieved Problems with Physical Activity, pre**	14.08	4.62	0.48*	-0.11	0.13	0.14	-0.06	-0.31*	1.00*																			
**8.**	**Sleep Disturbance, pre**	84.99	63.41	0.13*	-0.12	0.32*	0.17*	0.19*	-0.06	0.29*	1.00*																		
**9.**	**Problems with Social Support, pre**	10.98	3.14	0.03	-0.09	0.16*	0.09	0.17*	0.04	0.00	0.05	1.00*																	
**10.**	**Fear of Recurrence, pre**	7.81	2.27	0.10	-0.03	0.37*	0.31*	0.56*	0.16	-0.08	0.12	0.11	1.00*																
**11.**	**Depressive Symptoms, pre**	6.48	3.56	0.25*	-0.36*	0.41*	0.34*	0.20*	-0.06	0.22*	0.20*	0.32*	0.14	1.00*															
**12.**	**Condition**	0 = Control/Male		0.00	0.08	0.05	-0.05	0.01	-0.06	0.07	0.10	0.15	-0.12	0.02	1.00*														
**13.**	**Sex**	1= CBT/Female		0.00	-0.03	0.05	0.08	0.09	-0.05	0.00	0.16	0.02	0.07	0.00	-0.11	1.00*													
**14.**	**Age**	48.69	9.57	-0.11	-0.11	-0.14	-0.12	0.06	-0.12	0.07	0.11	-0.06	-0.07	-0.07	0.03	0.34*	1.00*												
**15.**	**Time since Treatment**	46.34	41.83	0.10	0.02	-0.03	-0.11	-0.21*	-0.01	-0.03	-0.12	0.11	-0.18*	-0.07	0.12	-0.22*	-0.06	1.00*											
**16.**	**Fatigue, post**	32.83	13.51	0.31*	-0.22*	0.12	0.17*	0.03	-0.09	0.08	0.16*	-0.02	0.09	0.19*	-0.59*	0.08	-0.02	0.01	1.00*										
**17.**	**Self-efficacy, post**	20.86	4.10	-0.20*	0.44*	-0.09	-0.15	-0.12	0.02	-0.08	-0.11	-0.10	-0.07	-0.26*	0.61*	-0.07	-0.06	0.00	-0.71*	1.00*									
**18.**	**Fatigue Catastrophizing, post**	17.18	5.80	0.16*	-0.21*	0.42*	0.29*	0.23*	0.02	-0.04	0.09	0.06	0.14	0.24*	-0.49*	0.28	-0.01	0.02	0.62*	-0.54*	1.00*								
**19.**	**Focusing on Symptoms, post**	22.41	9.43	0.17*	-0.21*	0.30*	0.46*	0.23*	0.05	0.04	0.13	0.00	0.19*	0.22*	-0.51*	0.09	-0.13	0.01	0.69*	-0.56*	0.67*	1.00*							
**20.**	**Problems Coping with Cancer, post**	9.19	11.41	0.04	-0.13	0.33*	0.33*	0.62*	0.00	-0.03	0.20*	0.06	0.45*	0.15	-0.09	0.10	0.05	-0.22*	0.22*	-0.26*	0.40*	0.38*	1.00*						
**21.**	**Physical Activity, post**	70.92	20.23	-0.15*	0.05	0.07	-0.04	0.11	0.56*	-0.29*	-0.04	-0.04	0.15	-0.07	0.13	-0.06	0.05	-0.01	-0.20*	0.15*	-0.05	-0.06	-0.01	1.00*					
**22.**	**Percieved Problems with Physical Activity, post**	10.52	4.91	0.30*	-0.24*	0.10	0.18*	0.00	-0.25	0.37*	0.08	-0.08	0.00	0.23*	-0.40*	0.14	0.09	0.06	0.70*	-0.48*	0.39*	0.50*	0.12	-0.45*	1.00*				
**23.**	**Sleep Disturbance, post**	43.11	51.57	0.16*	-0.17*	0.14	0.15	0.07	-0.10	0.12	0.28*	0.14*	0.11	0.22*	-0.45*	0.13	-0.03	-0.03	0.49*	-0.46*	0.45*	0.53*	0.14*	-0.16*	0.37*	1.00*			
**24.**	**Problems with Social Support, post**	10.41	3.27	0.13*	-0.04	0.20*	0.05	0.08	-0.03	0.02	0.10	0.55*	0.05	0.22*	-0.08	0.06	-0.11	0.10	0.17*	-0.18*	0.20*	0.07	0.04	-0.02	0.05	0.18*	1.00*		
**25.**	**Fear of Recurrence, post**	6.65	2.03	0.08	-0.04	0.30*	0.27*	0.48*	0.16	-0.07	0.09	0.06	0.74*	0.13	-0.24*	0.24	-0.04	-0.18*	0.23*	-0.21*	0.32*	0.32*	0.52*	0.07	0.10	0.22*	0.17*	1.00*	
**26.**	**Depressive Symptoms, post**	4.40	3.79	0.15	-0.26*	0.24*	0.21*	0.21*	0.05	0.09	0.16*	0.24*	0.11	0.61	-0.36*	0.12	0.00	-0.02	0.64*	-0.57*	0.51*	0.50*	0.31*	-0.09	0.49*	0.46*	0.32*	0.27*	1.00*

S2b Means, Standard Deviations [SDs] and correlation matrix showing the pairwise correlations between the different variables *without* z-score transformation. Polychoric correlation was used to compute the correlation between the dichotomous variables Condition and Sex. Polyserial correlation was used for pairs of either Condition or Sex with other variables. Pearson correlation was used for the remaining variable pairs. * indicates a significant correlation ($p < 0.05$). pre = pre-assessment, post = post-assessment.

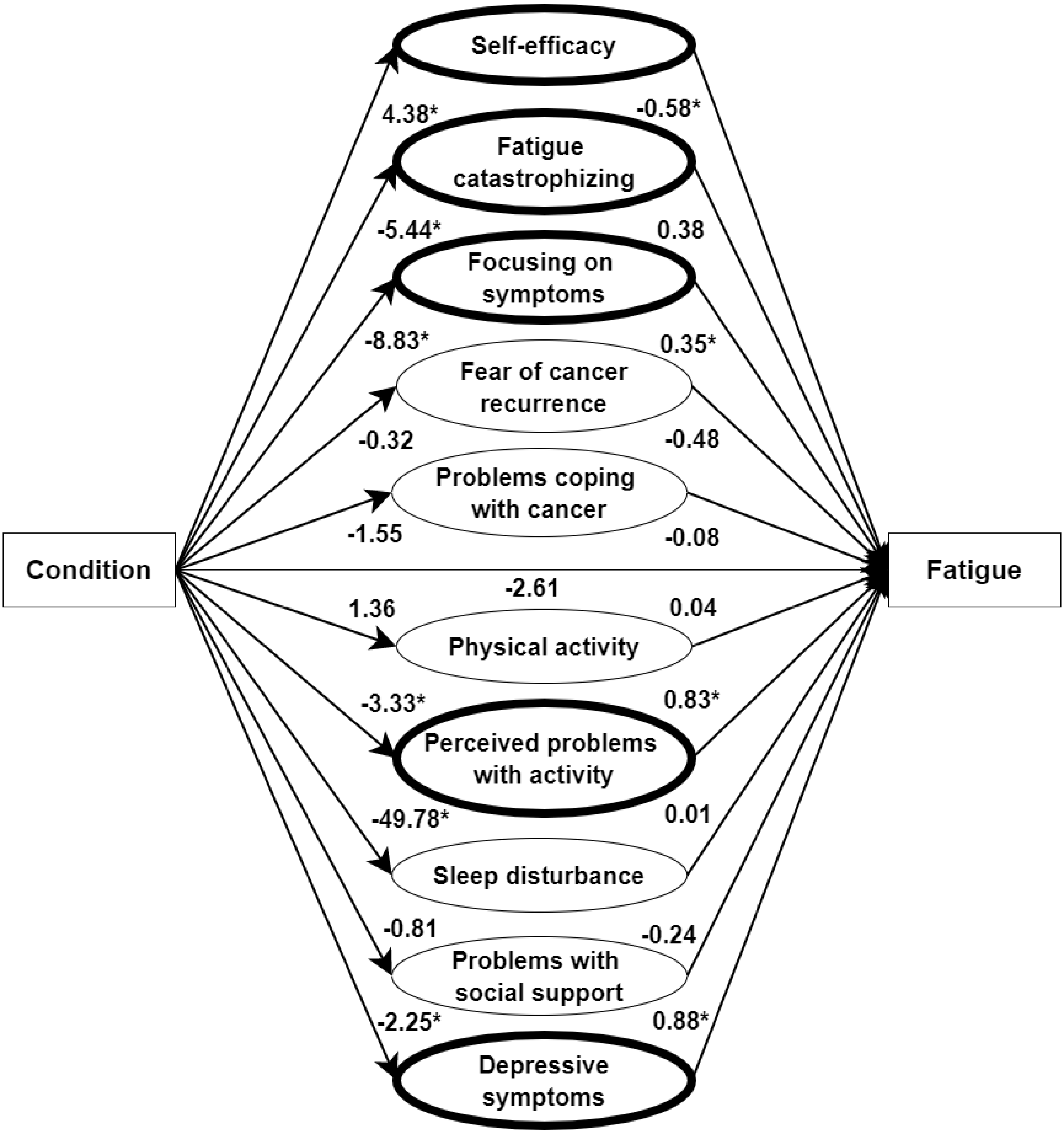

FIGURE S3 Classical mediation model: Sensitivity analysis *without* z-score transformation. Note: Even though the path from Fatigue catastrophizing to Fatigue is insignificant, the indirect effect from Condition to Fatigue through Fatigue catastrophizing is significant (*ab* = -2.06, CI[-4.68, -0.07]). * indicates a significant path.

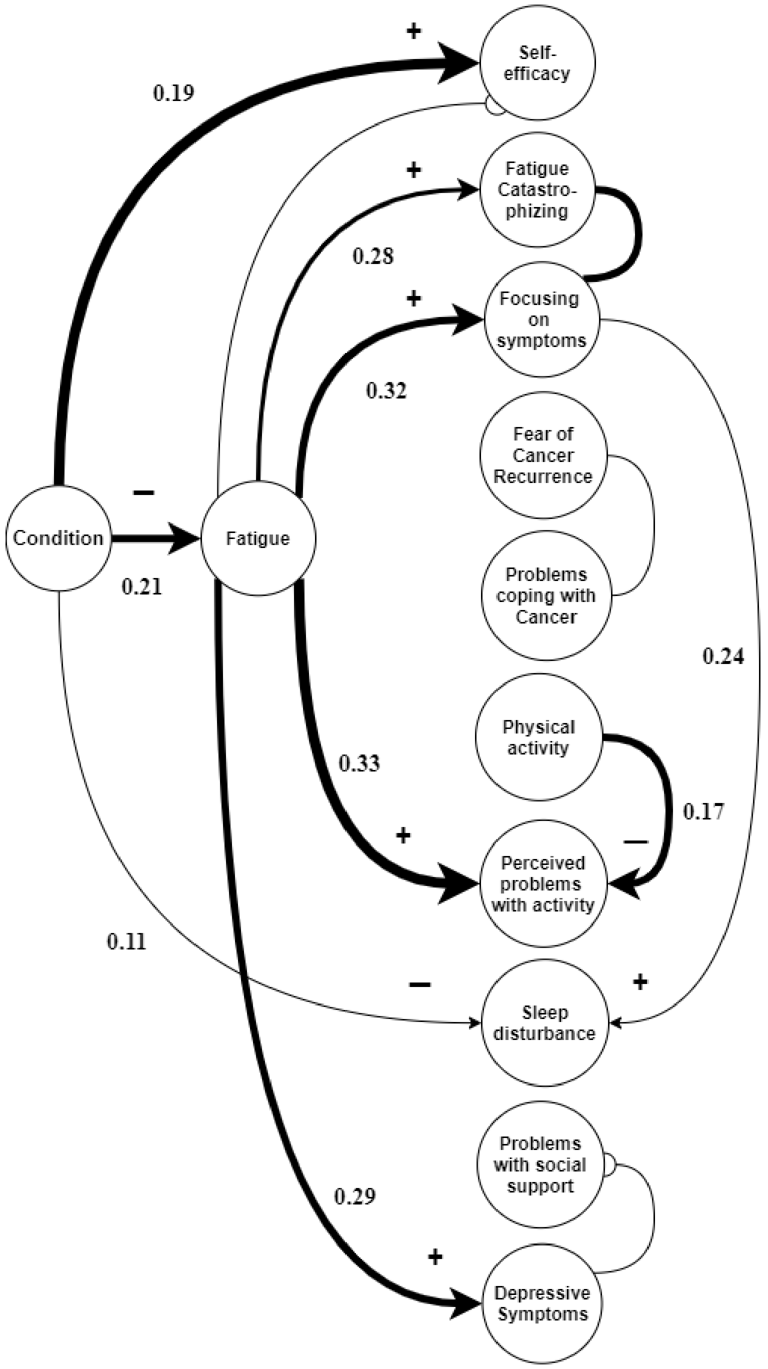

FIGURE S4 Causal discovery sensitivity analysis *without* z-score transformation.

Values in which different questionnaire(-versions) have been used are treated as missing values. The tail (-) represents the origin of the causal effect and the arrowhead (➤) the direction of the causal effect. The circle (o) represents an association in which the origin and direction are unclear. The undirected lines (–) indicate the presence of selection bias (i.e. bias introduced by the sample selection).
All links represent a causal association of which the edge has a post-bootstrap reliability coefficient of ≥ 0.5, with a thicker line corresponding to a more likely causal association between variables. The values represent the strength of the causal effects (see also Table S2b).

References

1. Gielissen MFM, Verhagen CAHHVM, Bleijenberg G. Cognitive behaviour therapy for fatigued cancer survivors: long-term follow-up. Br J Cancer. 2007;97(5):612–8. https://doi.org/10.1038/sj.bjc.6603899
2. Jacobsen PB, Azzarello LM, Hann DM. Relation of Catastrophizing to Fatigue Severity in Women with Breast cancer. Cancer Res Ther Control. 1999;8:155–64.
3. Van Damme S, Crombez G, Bijttebier P, Goubert L, Van Houdenhove B. A confirmatory factor analysis of the Pain Catastrophizing Scale: Invariant factor structure across clinical and non-clinical populations. Pain. 2002;96(3):319–24.
4. Ray C, Weir W, Stewart D, Miller P, Hyde G. Ways of coping with Chronic Fatigue Syndrome: Development of an illness management questionnaire. Soc Sci Med. 1993;37(3):385–91.
5. Servaes P, Verhagen S, Schreuder HWB, Veth RP., Bleijenberg G. Fatigue after treatment for malignant and benign bone and soft tissue tumors. J Pain Symptom Manage. 2003;26(6):1113–22.
6. Brom D, Kleber RJ. De Schok Verwerkings Lijst [The Dutch version of the Impact of Event Scale]. Ned Tijdschr voor Psychol. 1985;40:164–8.
7. Horowitz M, Wilner N, Alvarez W. Impact of Event Scale: A Measure of Subjective Stress. Psychosom Med. 1979;41(3):209–18.
8. Van der Werf SP, Prins JB, Vercoulen JHMM, Van der Meer JWM, Bleijenberg G. Identifying physical activity patterns in chronic fatigue syndrome using actigraphic assessment. J Psychosom Res. 2000;49(5):373–9.
9. Vercoulen JHMM, Swanink CMA, Fennis JFM, Galama JMD, van der Meer JWM, Bleijenberg G. Dimensional assessment of chronic fatigue syndrome. J Psychosom Res. 1994;38(5):383–92.
10. Bergner MB, Bobbitt RA, Carter WB, Gilson BS. The Sickness Impact Profile: Development and Final Revision of a Health Status Measure. Med Care. 1981;19(8):787–805.
11. Van Sonderen E. Het meten van sociale steun met de Sociale Steun Lijst-Interacties (SSL-I) en Sociale Steun Lijst-Discrepanties (SSL-D): een handleiding [Measuring social support with the Sonderen Social Support Inventory: instruction manual]. Groningen, The Netherlands: Noordelijk Centrum voor Gezondheidsvraagstukken, Rijksuniversiteit Groningen; 1993.
12. Zigmond AS, Snaith RP. The Hospital Anxiety and Depression Scale. Acta Psychiatr Scand. 1983;67:370–367. https://doi.org/10.1111/j.1600-0447.1983.tb09716.x
13. Beck AT, Guth D, Steer RA, Ball R. Screening for major depression disorders in medical inpatients with the Beck Depression Inventory for Primary Care. Behav Res Ther. 1997;35(8):785–91.
14. Cohen J. A Power Primer. Psychol Bull. 1992;112(1):155–9. https://doi.org/10.1038/141613a0
15. Kass RE, Raftery AE. Bayes Factors. J Am Stat Assoc. 1995;90(430):773–95.
